# Insurance-based risk-sharing agreements

**DOI:** 10.1186/2052-3211-8-S1-P22

**Published:** 2015-10-05

**Authors:** Augustin Terlinden, Amine Aissaoui, Olivier Ethgen

**Affiliations:** 1 Blue Antidote, Brussels, 1050, Belgium; 2 Paris Dauphine University, Paris, 75016, France; 3 University of Liège, Liège, 4000, Belgium

## 

Stretched healthcare budgets have been tensing up patient access negotiations between healthcare payers and manufacturers. Data and the associated evidence available at registration are often deemed insufficient to accurately estimate the real-life clinical outcomes and budget impact. Payers want to reduce budget uncertainty and manufacturers need to evolve in a competing healthcare environment.

Risk-sharing agreements (RSAs) are on the rising trend. Conceptually, RSAs have the remarkable advantage of reducing payer exposure to the financial risks associated with the introduction of a new healthcare intervention. However, engaging in a RSA should be cautiously thought through and planned as those contracts entail important financial implications, notably for the manufacturer. Monitoring costs are elevated and might jeopardize the implementation[1].

Nowadays, most of the current RSAs tend to shift the uncertainty around an expected outcome from the healthcare payer to the manufacturers. Although one cannot refer to risk-sharing per se, manufacturers use it during negotiations as an alternative to price reduction[2].

**Figure 1:**
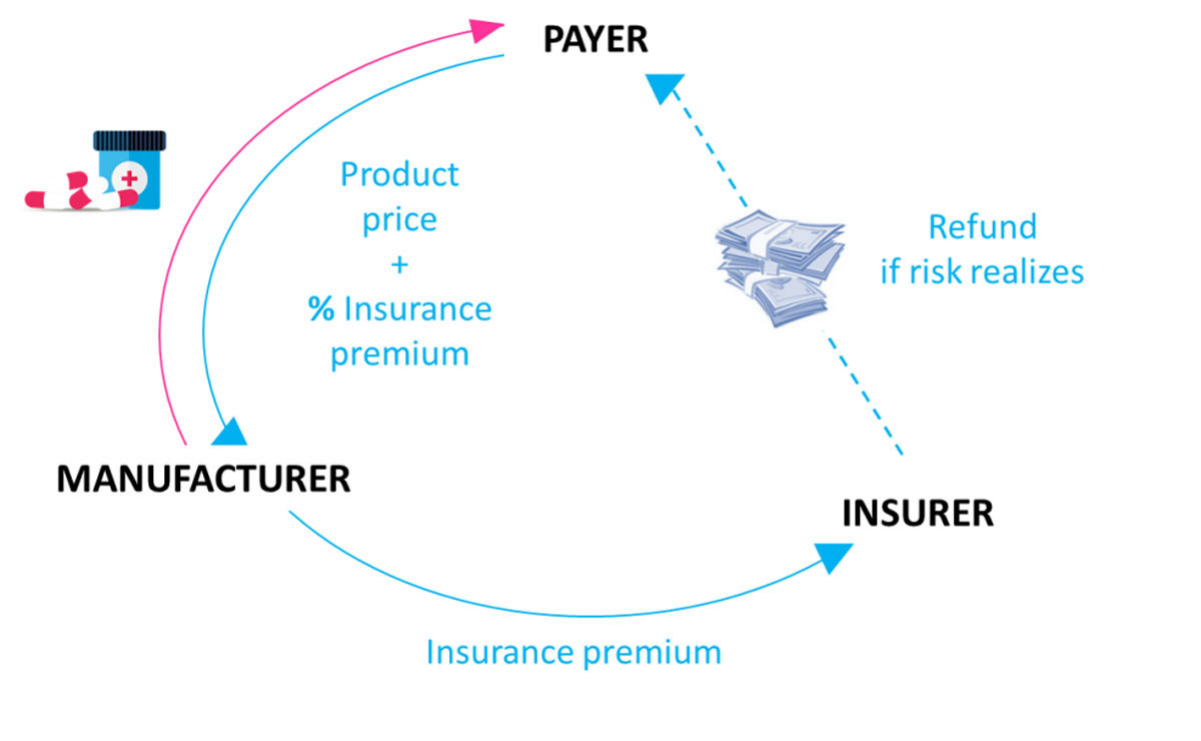
The risk embedded in healthcare contract did not simply shift from the payer to the manufacturer. An insurer stands behind the manufacturer. It reimburses the payer if the risk realizes

**Figure 2:**
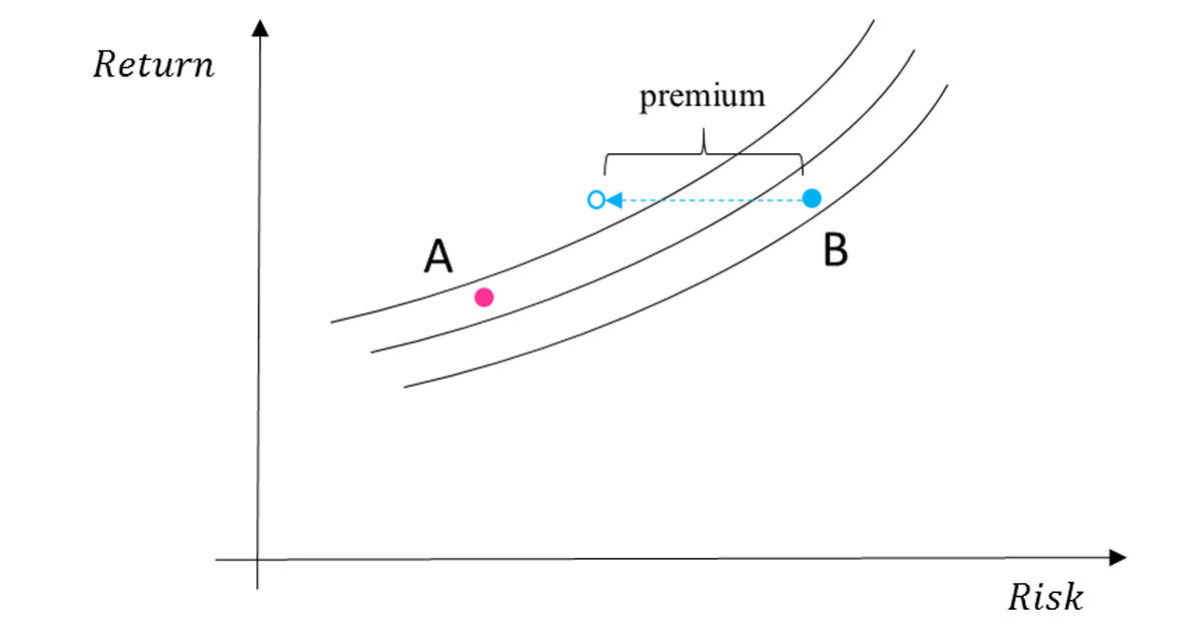
When packaged with an insurance contract, treatment B is preferred to treatment A.

We will define the insurance approach in the staggered financial evaluation of a potential RSA. The risk will be quantified from the point of view of three professional risk-takers (i.e. an investment bank, a reinsurer and a pension fund) willing to insure the transaction (Figure 1). Various formulas to split this risk-equivalent cost (i.e. insurance premium) between a manufacturer and a payer will be proposed (Figure 2). Budget gains for switching from the actual situation to the three risk-equivalent costs methodologies will be illustrated using a fictive oncology product.
